# ABCG2 transporter reduces protein aggregation in cigarette smoke condensate-exposed A549 lung cancer cells

**DOI:** 10.1371/journal.pone.0297661

**Published:** 2024-03-05

**Authors:** Emmanuella O. Ajenu, Ashley M. Seideneck, Esh Pandellapalli, Emily M. Shinsky, Casey L. Humphries, Nicholas L. Aparicio, Mahak Sharma, James H. Marden, Maria M. Krasilnikova

**Affiliations:** 1 Department of Biochemistry and Molecular Biology, Penn State University, University Park, Pennsylvania, United states of America; 2 Department of Biology, Penn State University, University Park, Pennsylvania, United states of America; Sichuan University West China Hospital, CHINA

## Abstract

Cigarette smoke-induced protein aggregation damages the lung cells in emphysema and COPD; however, lung cancer cells continue to thrive, evolving to persist in the toxic environment. Here, we showed that upon the cigarette smoke condensate exposure, A549 lung cancer cells exhibit better survival and reduced level of protein aggregation when compared to non-cancerous Beas-2B and H-6053 cells. Our data suggests that upregulation of efflux pumps in cancer cells assists in reducing smoke toxicity. Specifically, we demonstrated that inhibition of the ABCG2 transporter in A549 by febuxostat or its downregulation by shRNA-mediated RNA interference resulted in a significant increase in protein aggregation due to smoke exposure.

## Introduction

Cigarette smoke (CS) contains several known carcinogenic compounds, such as polycyclic aromatic hydrocarbons (PAHs), in addition to reactive oxidative and nitrogen species (ROS and RNS) [1–4]. Thus, it represents a frequent etiological agent for the development and progression of chronic obstructive pulmonary disease (COPD) and lung cancer [5–7]. Upon CS exposure, the cellular detoxification system faces an increased burden due to the large influx of reactive species released from the cigarette combustion. Inability of antioxidant enzymes to offset the damage from CS can lead to a state of oxidative stress that affects multiple components of the cells including lipids, proteins, and DNA [8–10].

Oxidative stress leads to an increase in the amount of misfolded, ubiquitinated proteins, which can also result in formation of intracellular protein aggregates, indicative of a proteostatic imbalance [11]. CS-impaired autophagy has been suggested as a plausible mechanism for the formation of these protein aggregates, known as aggresomes [12]. Protein aggregation has been implicated in cell injury and death [13]; it has been particularly well established in certain neurodegenerative pathologies [14], such as amyotrophic lateral sclerosis (ALS), Alzheimer’s, and Parkinson’s [15]. It has also been shown that aggregation of P53 has a role in cancer progression [16]. CS exposure was shown to interfere with autophagy, resulting in protein aggregation in emphysema and COPD [12].

However, CS-induced protein aggregation in lung cancer has not been extensively studied. Cancer cells undergo selection processes that allows the development of cells that are well adjusted to a specific environment [17]. We suggest that the lung cancer cells develop a mechanism that allows them to adapt to toxicity of CS, similar to their adjustment to cancer treatments. Upregulation of efflux pumps is a common mechanism of multidrug resistance in cancer [18].

Here, we tested a hypothesis that an efflux pump can decrease the protein aggregation caused by CS in cancer cell line A549. Specifically, we focused on the role of ATP-binding cassette G2 (ABCG2) [19], which has been previously shown to be highly expressed in some lung cancer cell lines, including an adenocarcinoma cell line A549 [20].

ABCG2, also known as breast cancer resistance protein (BCRP), is a transmembrane protein of 655 amino acids that has an extensive substrate profile [21], with known substrates including chemotherapeutics, endogenous metabolites, and their derivatives [22]. ABCG2’s role is widely known for conferring multidrug resistance in cancer [23, 24], acting as an efflux pump for anti-cancer medications, such as methotrexate, topotecan, and mitoxantrone [25]. Besides anticancer medications, ABCG2 is upregulated by glucocorticoids, HDAC inhibitors, and some non-coding RNAs [26, 27]. ABCG2 substrates are mostly polycyclic and hydrophobic molecules [28], which makes it a plausible candidate for pumping out some of the polycyclic aromatic hydrocarbons, known to be contained in CS [29]. In line with that, ABCG2 was shown to be upregulated in some lung cancer cells, including A549, in response to smoke exposure [20].

In this study, by decreasing the activity of ABCG2 pump in A549, we showed that it has a protective effect against CS-induced protein aggregation, likely by the efflux of toxic components of CS. Inhibition of ABCG2 by chemical and genetic methods yielded a significant increase in the intracellular concentration of protein granules in A549 cells upon exposure to cigarette smoke condensate (CSC).

## Materials and methods

### Mammalian cell growth

Human lung adenocarcinoma cell line A549, human non-tumorigenic lung epithelial cell line Beas-2B, and CV1 monkey kidney cells were obtained from American Type Culture Collection (ATCC). A549 cells were cultured in F-12 Kaighn’s Modification Medium, supplemented with 10% (v/v) fetal bovine serum (FBS). Beas-2B cells were grown in Bronchial Epithelial Cell Growth Medium (BEGM) BulletKit (Lonza) supplemented with 10% (v/v) FBS, and CV1 cells in DMEM media, supplemented with 10% (v/v) FBS. Human primary alveolar epithelial H-6053 cells (Cell Biologics) were grown in Epithelial Cell medium with Kit (Cell Biologics). All cell lines were grown in a 37 °C incubator with 5% CO2 atmosphere.

### Cigarette smoke condensate preparation

Research cigarettes 1R6F were purchased from the University of Kentucky Center for Tobacco Reference Products. Cigarette smoke condensate (CSC) was obtained by passing the cigarette smoke from one cigarette through a Whatman filter paper fixed in a vacuum unit, followed by elution with 1 ml DMSO. CSC was added to the media used in cell growth; specific amounts and incubation times varied among experiments. Control samples were treated with amounts of DMSO, equal to those introduced with CSC.

### Cell counting for growth analysis

Cells were grown in 35 mm dishes in 2 ml of their corresponding media, supplemented with 0.3 μl, 1 μl, 3 μl, or 9 μl of CSC, or DMSO as a control, with daily replacement of the growth media. The cells’ images were taken every day using Evos Cell Imaging System 4x objective. The cells were counted within 3–8 screen images throughout the area of culture dish, depending on the extent of clustering in the cultures, and the average numbers were plotted. The size of a screen for the 4x objective roughly corresponded to the 15.2 mm^2^ area of a tissue culture dish. The cell counting was performed using Fiji software.

### ABCG2 inhibition by febuxostat

ABCG2 inhibitor febuxostat (TCI) was first dissolved in DMSO at a concentration of 10 g/L, then diluted an additional 100 times with water prior to adding to the cells. A549 cells were grown in 35 mm dishes with glass coverslips, containing 2 ml media in the presence of 1 μg/ml febuxostat for 20 hours. Since febuxostat was initially dissolved in DMSO, the corresponding amount of DMSO was added to control.

### ABCG2 knockdown by RNAi

To knockdown ABCG2, A549 cells were infected with lentiviruses that expressed shRNAs, targeted to the ABCG2 transcript (5’-GGAGGCAAATCTTCGTTATTA-3’) or a non-targeting sequence (5’- TCTCGCTTGGGCGAGAGTAAG -3’). The transfer plasmids were obtained by inserting the annealed oligonucleotides, containing shRNA sequences, into the XhoI and HpaI restriction sites of pLL 3.7 [30]. pLL3.7 was a gift from Luk Parijs (Addgene plasmid # 11795); pMD2.G and psPAX2 were gifts from Didier Trono (Addgene plasmid # 12259, # 12260).

Lentiviruses were obtained by transfecting 293T cells in a 10 cm dish with 2 μg pMD2.G, 4 μg psPAX2 [31], and 8 μg of the transfer plasmid, in a 1:3 ratio with polyethyleneimine [32]. Media collected at 48 and 72 hours after transfection were combined. A549 cells were transduced with 2:1 mix of collected medium and A549 growth medium, supplemented with 10 μg/ml DEAE-Dextran for 24 hours. The cells were stained, and the mRNA was isolated on the 5th day after infection, with or without CSC exposure. The efficiency of transduction was calculated as a ratio of GFP-expressing to total number of cells. The amount of ABCG2 transcript was analyzed by RT-PCR. To calculate the efficiency of the knockdown, the amount of ABCG2 transcript in shABCG2-containing cells was first normalized by its amount in shNT-containing cells, subtracted from one, and multiplied by 100.

### Detection of protein aggregation

Cells were incubated for the indicated amount of time, with various concentrations of CSC. For more than 24 hours incubation with CSC, the media containing CSC was refreshed every other day. For the detection of protein aggregation, the cells were stained with Proteostat Aggresome Detection Kit (Enzo) according to the manufacturer’s protocol.

Images were obtained with Zeiss LSM 880 confocal microscope at 63x magnification with oil immersion at excitation/emission wavelengths of 500/600 nm. Quantitative analysis of aggregation was performed as described below, using Fiji software. The mean fluorescence per unit area of the cytoplasm, normalized by the same in the space between the cells (NFI), was calculated for 10–30 cells across three fields of view; data represent the mean ± standard error. The experiments were repeated three times. Statistical analysis was performed using the Mann-Whitney U-Test (**** P value <0.0001, *** P value< 0.001, ** P value< 0.01, ns = not significant).

### Real time RT-PCR

RNA was isolated from cells grown in 35 mm dishes using silica columns [33]. Reverse transcription reactions were performed using qScript cDNA synthesis kit (Quantabio) with 500 ng of RNA per reaction, according to manufacturer’s instructions. The cDNAs were then precipitated with ethanol and used for qPCR with PerfeCTa SYBR Green SuperMix reagent (Quantabio) on a StepOne^™^ Real-Time PCR System, according to their recommended procedures.

#### Primers (5′-to-3′) used in real time PCR

ABCG2-forward  GGAGGCCTTGGGATACTTTGA
ABCG2-reverse   GCTCTATGATCTCTGTGGCTTTA
GAPDH-forward GAAGGTCGGAGTCAACGG
GAPDH-reverse  ATGGCAACAATATCCACTTTACC

### Western blot

Cells were grown in 35 mm dishes with addition of 3μl or 6 μl of CSC for indicated periods of time (1–24 hours), and lysed using Transmembrane Protein Extraction Reagent (Fivephoton Biochemicals), according to the manufacturer’s protocol. Then, 13 μl of each sample was mixed with 5 μl 4x Laemmli buffer (BioRad) and 2 μl 1M DTT (Dithiothreitol), and denatured at 37°C for 30 minutes. The samples were then separated in 8% SDS-PAGE gel and transferred to 0.45 μm PVDF membrane (Thermo Fisher Scientific) using Semidry Electroblotting System (Owl) at 2mA/cm^2^ for 2 hours in Tris-Glycine-SDS transfer buffer (Sigma-Aldrich) with 20% methanol. Then, the membranes were dried for 30 minutes, blocked with Blocking solution (TBST, Tris-Buffered Saline with Tween 20 (Sigma-Aldrich), supplemented with 5% v/w powdered milk (Carnation)) at room temperature for 1 hour, and incubated overnight at for 4°C with primary antibodies diluted in the Blocking solution. Mouse ABCG2 Antibody (B-1) (Santa Cruz SC-377176) and rabbit GAPDH (Thermo Fisher Scientific, PA1-987) were used as primary antibodies; ABCG2 antibody was diluted 1:200, and GAPDH was diluted 1:1000 in Blocking solution. The membranes were washed three times in TBST, and then incubated with secondary antibodies, diluted 1:5000 in Blocking solution, for one hour at room temperature. Secondary goat anti-rabbit HRP-conjugated antibody (Thermo Fisher Scientific, 31460) was used for detection of GAPDH, and anti-mouse IgGκ BP-HRP (Santa Cruz, sc-516102) was used for detection of ABCG2. The membranes were washed three times with TBST, and the proteins were detected by incubation with Western Chemiluminescent HRP Substrate (Immobilon), according to the producer’s protocol, followed by X-ray film exposure.

## Results

### Reduced protein aggregation in lung cancer cell line A549 compared to normal monkey kidney cell line CV1

Previous studies demonstrated protein aggregation in patients with emphysema and COPD, which showed that the normal lung tissue is strongly affected by CS. In our studies, we focused on the effects of smoke on cancer cell line A549. We hypothesized that some mechanisms counteracting frequent smoke exposure may exist in lung cancer cell lines.

To preliminary test this hypothesis, we first compared protein aggregation caused by smoke exposure in human lung cancer cell line A549 to a normal mammalian cell line CV1, originating from the monkey kidney.

Using a commercially available Proteostat Aggresome Detection Kit, we evaluated the degree of global protein aggregation, as denoted by the formation of distinct granules. We benchmarked this study using a known inhibitor of the proteasome and inducer of protein aggregation, MG132. A549 cells were treated with either 10 μM of MG132 for 18 hours or with a DMSO vehicle control. As shown in S1 Fig, MG132 induced global protein aggregation throughout the cytoplasm of A549 cells whereas minimal granularity of the fluorescent probe was detected in the DMSO-treated cells.

**Fig 1:**
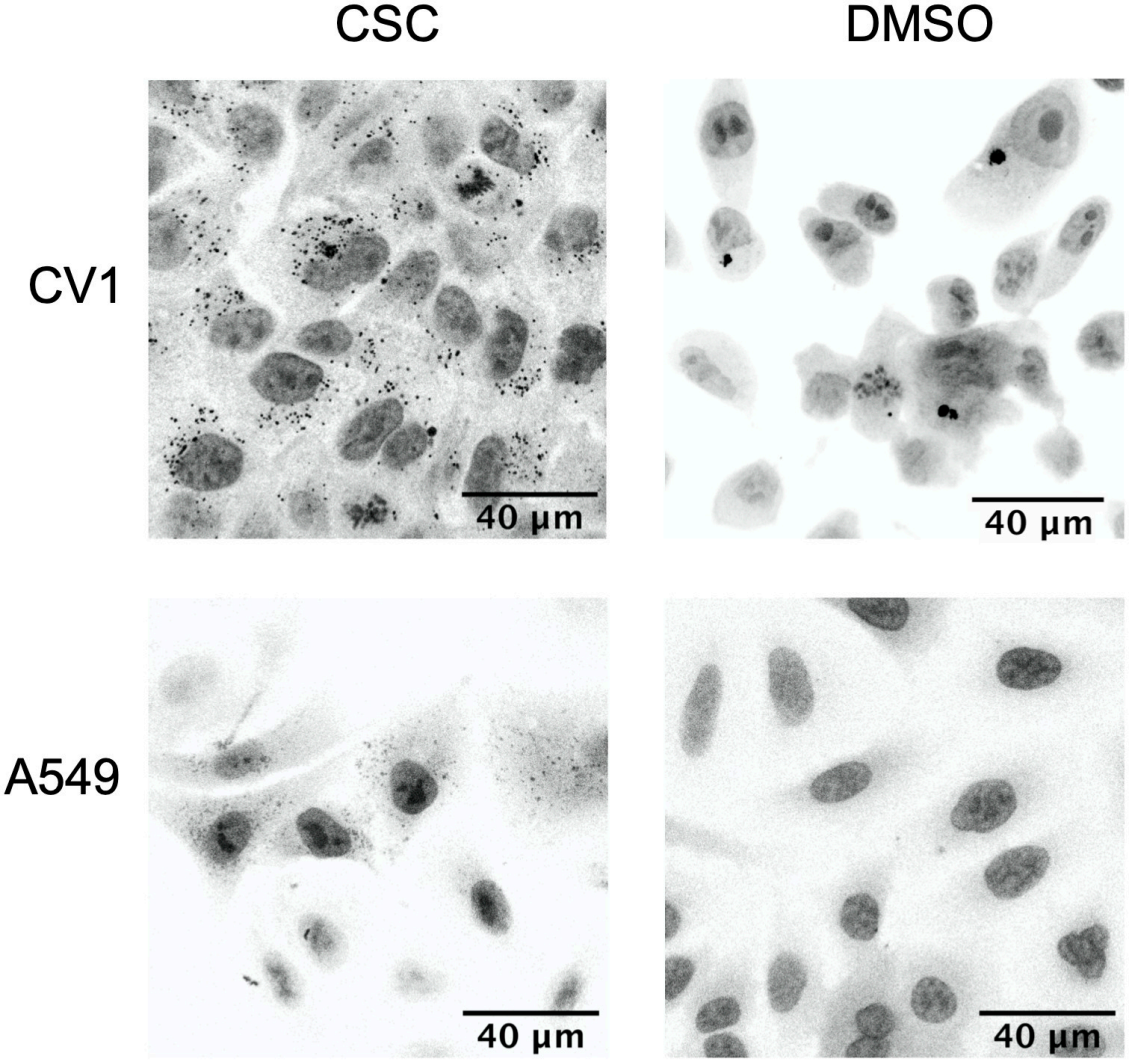
Protein aggregation caused by CSC exposure is less in cancer cells A549, than normal monkey kidney CV1 cells. The cells were grown in the presence of 0.3 μl of CSC for 24 hours, then stained with Proteostat Aggresome Detection kit.

Next, we compared CSC-induced protein aggregation in A549 and CV1. Upon exposure to CSC for 24 hours, granule formation was more pronounced in the cytoplasm of normal kidney CV1 cells as compared to the A549 lung cancer cell line (Fig 1). This result prompted us to evaluate the protein aggregation in a non-cancerous human lung cell lines since the observed effect could be specific for the lung, and not associated with cancer.

### Protein aggregation in lung cancer cell line A549 is less than in normal lung cell lines Beas-2B and H-6053 across a range of concentrations of CSC

To determine whether the protein aggregation in A549 is also less than in a non-cancerous human lung cell lines, we chose to use lung alveolar Beas-2B cell line, which has been previously used as a model system to study protein aggregation due to tobacco smoke exposure [12], and normal lung alveolar cells H-6053.

**Fig 2:**
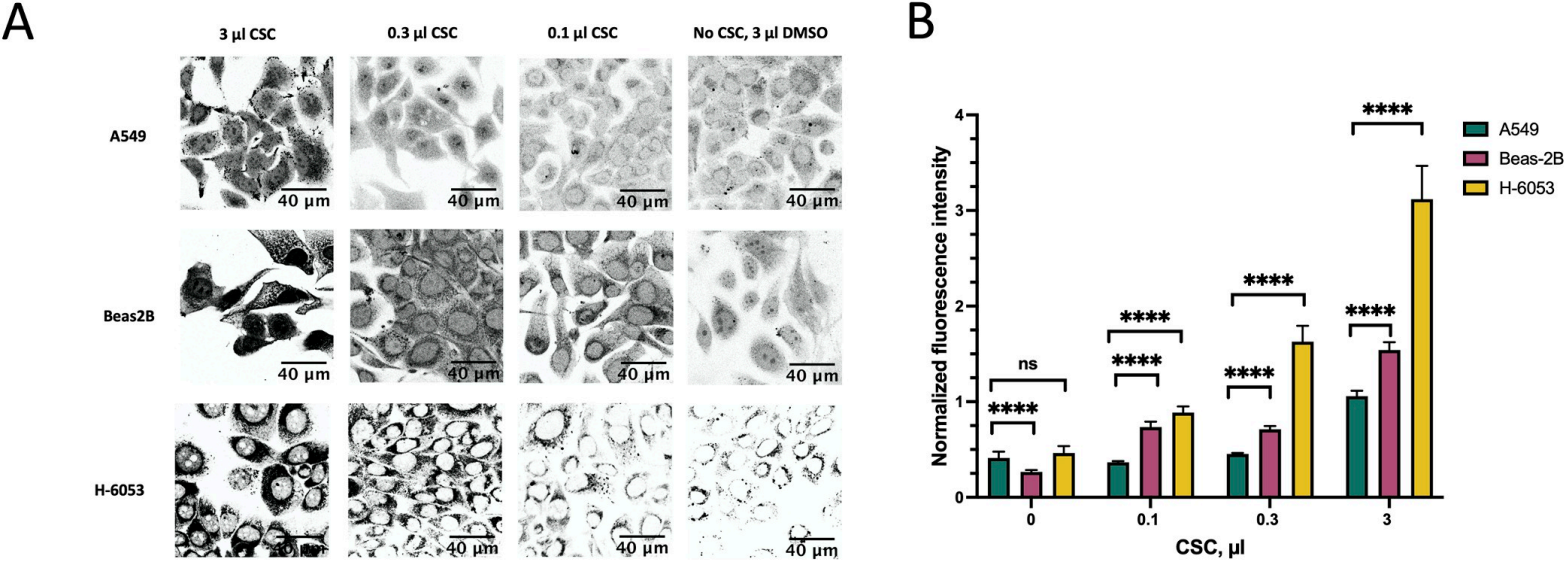
Comparison of protein aggregation in lung cancer cell line A549 and non-cancer cell lines Beas-2B and H-6053 across different CSC concentrations. The cells were exposed to different concentrations of CSC for 24 hours. A. Protein aggregates, stained by Proteostat Aggresome Detection kit. B. NFI of the protein aggregates shown in A.

To this end, we treated A549, Beas-2B, and H-6053 cells with increasing concentrations of CSC for 24 hours (Fig 2A). In order to quantitatively compare the levels of protein aggregation, we calculated the normalized fluorescence intensity (NFI), the mean fluorescence per unit area of the cytoplasm, normalized to the same value obtained from cell-free areas. NFI was higher in the non-cancerous Beas-2B and H-6053 than in the cancerous A549 cell line at all concentrations of CSC that we tested (Fig 2B).

The distinctive protein granules in Beas-2B and H-6053 cells appeared even in 0.1 μl CSC, however, in A549 they became obvious only at 3 μl CSC (Fig 2A). The increase in NFI upon 3 μl CSC exposure, compared to DMSO control, was about 2-fold for A549 and about 6-fold for Beas-2B and H-6053.

**Fig 3:**
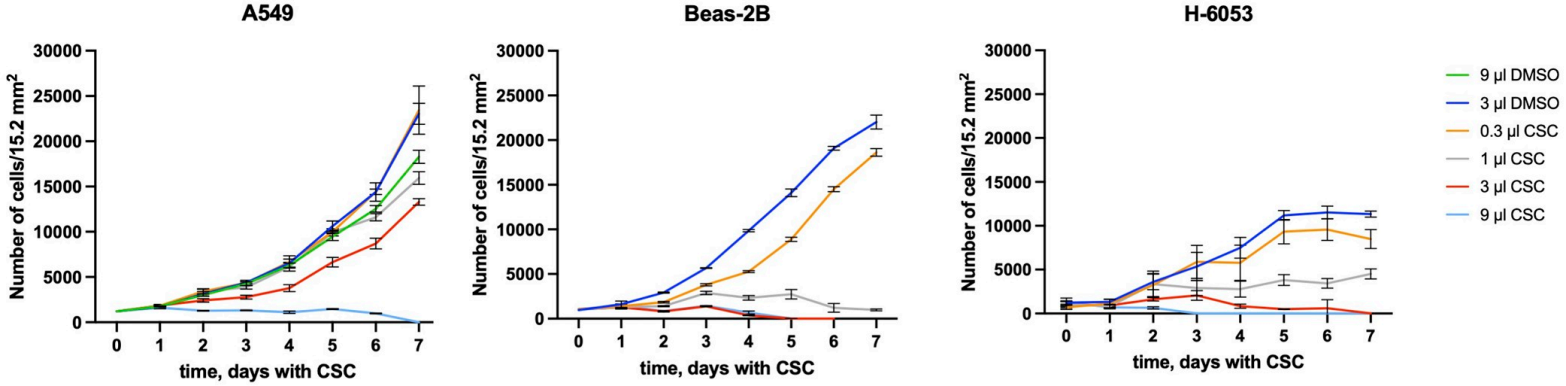
Growth curves of A549, Beas2B, and H-6053 in different concentrations of smoke. The time dependence of the average cell numbers in 15.2 mm^2^ area corresponding to the field of view of the microscope screen with 4x objective.

A549 had a higher level of aggregation than Beas-2B without smoke exposure, since the cancer cells are known to have increased level of protein aggregation, while H-6053 had the level comparable to A549. Based on our growth analysis, H-6053 cells grew at much slower rate and to less density than other cell lines (Fig 3); we believe that growth in tissue culture conditions is stressful for this primary alveolar epithelium cells, resulting in an increased protein aggregation.

### Cancer cell line A549 demonstrates better resilience under CSC exposure, compared to non-cancerous Beas-2B and normal lung H-6053 cell lines

Next, we explored whether the decreased protein aggregation in A549 cell line is also associated with its increased survival in the presence of CSC. To this end, we incubated the cells with different amounts of CSC (0.3 μl, 1.0 μl, 3.0 μl, 9 μl CSC, added to 2 ml of media), and DMSO (3 μl or 9 μl) as a control (Fig 3). After addition of CSC or DMSO, the cells were counted every day for 7 days, and the growth curves were plotted. We observed that A549 cells growth was unchanged in 0.3 μl of CSC, while they could still grow with reduced growth rates in up to 3 μl of smoke. They stopped growing only in 9 μl CSC per 2 ml of media. At this concentration of CSC, they were gradually decreasing in number, and eventually dying by day 7. To make sure that CSC, and not DMSO in which it was dissolved, caused the cell death, we included an extra control, with media supplemented with 9 μl DMSO. Interestingly, 9 μl DMSO (0.45%) also caused a reduced growth rate, compared to the one in 3 μl DMSO, but did not cause the cell death.

In contrast to cancer cell line A549, both non-cancerous cell lines were dying in much lower concentrations of CSC. They were able to grow with a reduced rate in 0.3 μl CSC. In 1 μl of CSC, they survived for at least seven days without much growth. In 3 μl CSC, they were deteriorating and dying.

Strikingly, the cancerous cell line A549 could maintain growth at about 10 times high concentration of CSC compared to non-cancerous lung cell lines (3 μl vs 0.3 μl of CSC).

### Protein aggregation in non-cancerous lung cell line Beas-2B and cancerous A549 during continuous CSC exposure

**Fig 4:**
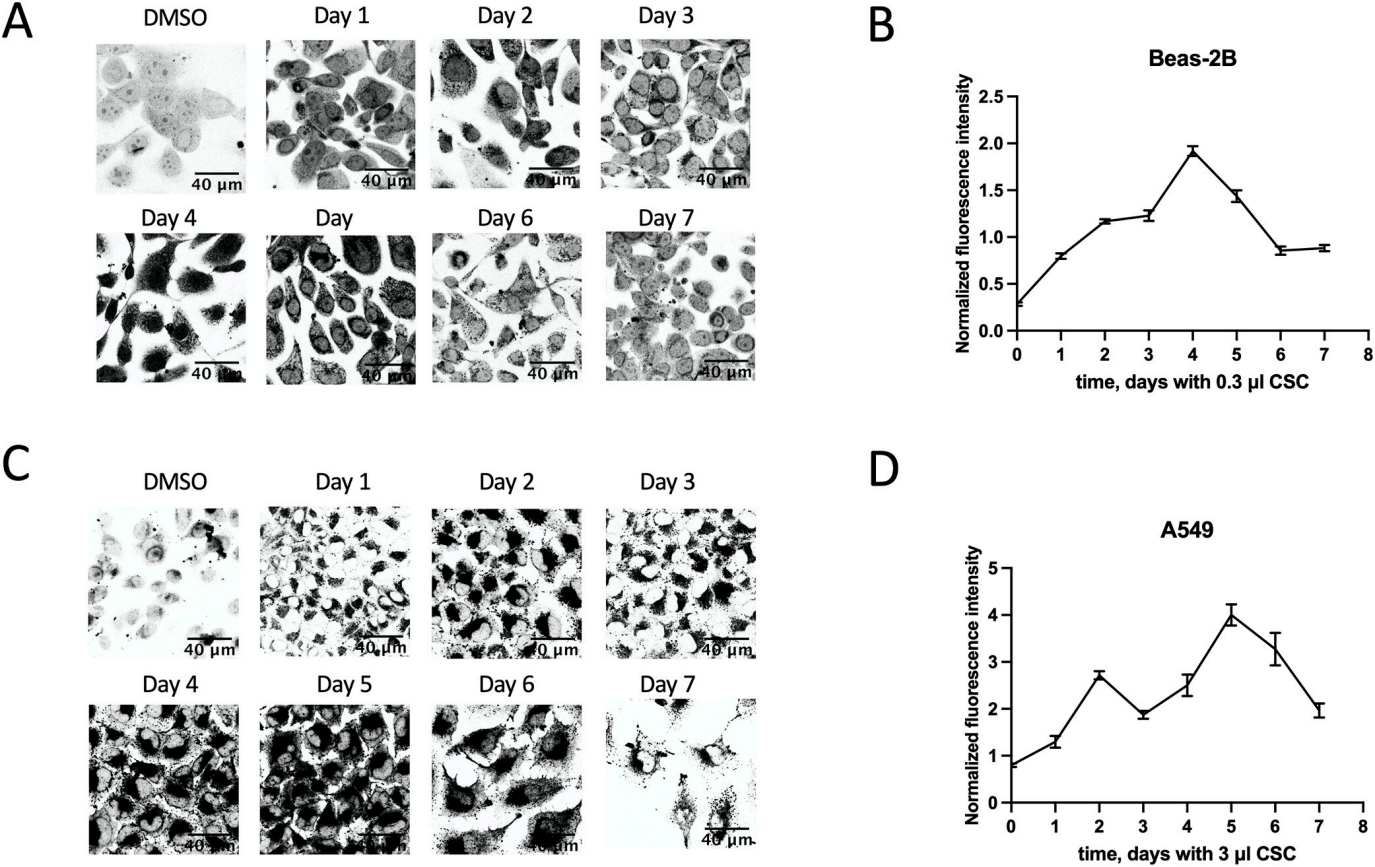
Time dependence of protein aggregation in lung cell lines. A. Protein aggregation in Beas-2B cells. Each cover slip was incubated with 0.3 μl of CSC per 2 ml of media for indicated number of days. Protein aggregates were stained by Proteostat Aggresome Detection kit. B. NFI of the protein aggregates shown in A. C. Protein aggregation in A549 cells, incubated with 3 μl of CSC per 2 ml of media. Note that the amount of CSC used for A549 is 10 times more than for Beas-2B. D. NFI of the protein aggregates shown in C.

To observe whether the Beas-2B and A549 lung cells can adapt to CSC over time, we performed a temporal study that looked at protein aggregation over seven days, with a continuous exposure to concentrations of CSC that allowed for a reduced growth rate (as determined from Fig 3). We used 0.3 μl CSC for Beas-2B (Fig 4A), and 3 μl CSC for A549 cells (Fig 4C).

The maximum fluorescence was observed for Beas-2B after 4 days of exposure, and thereafter decreased. In A549, the maximum fluorescence was reached by day 5, with a local maximum at 2 days.

We concluded that over the week time, we observed an adaptation to the CSC environment that allowed some aggregation decrease to occur from day 4–5 to day 7 in both Beas-2B and A549 (Fig 4B and 4D). By day 7, the aggregation still remained much higher than in a negative control in both cell lines, although we could not exclude that some additional reduction in aggregation may occur over the longer period of time.

### The expression of ABCG2 was significantly higher in A549 cancer cell line, comparing to non-cancer cells

We then probed what mechanistically might be resulting in the observed decrease of protein aggregation in A549 cells. One possibility was that an efflux pump was quickly removing some compounds found in CSC, thus preventing protein aggregation. We decided to focus on the ABCG2 transporter, which was known to be upregulated in A549 cells [20]. The choice of a specific transporter was based on our observations that live A549 cells were difficult to stain with Hoechst 33258. The activity of ABCG2 has been previously shown to be responsible for pumping out a Hoechst dye [34], which indicated that ABCG2 pump could be highly active in A549.

**Fig 5:**
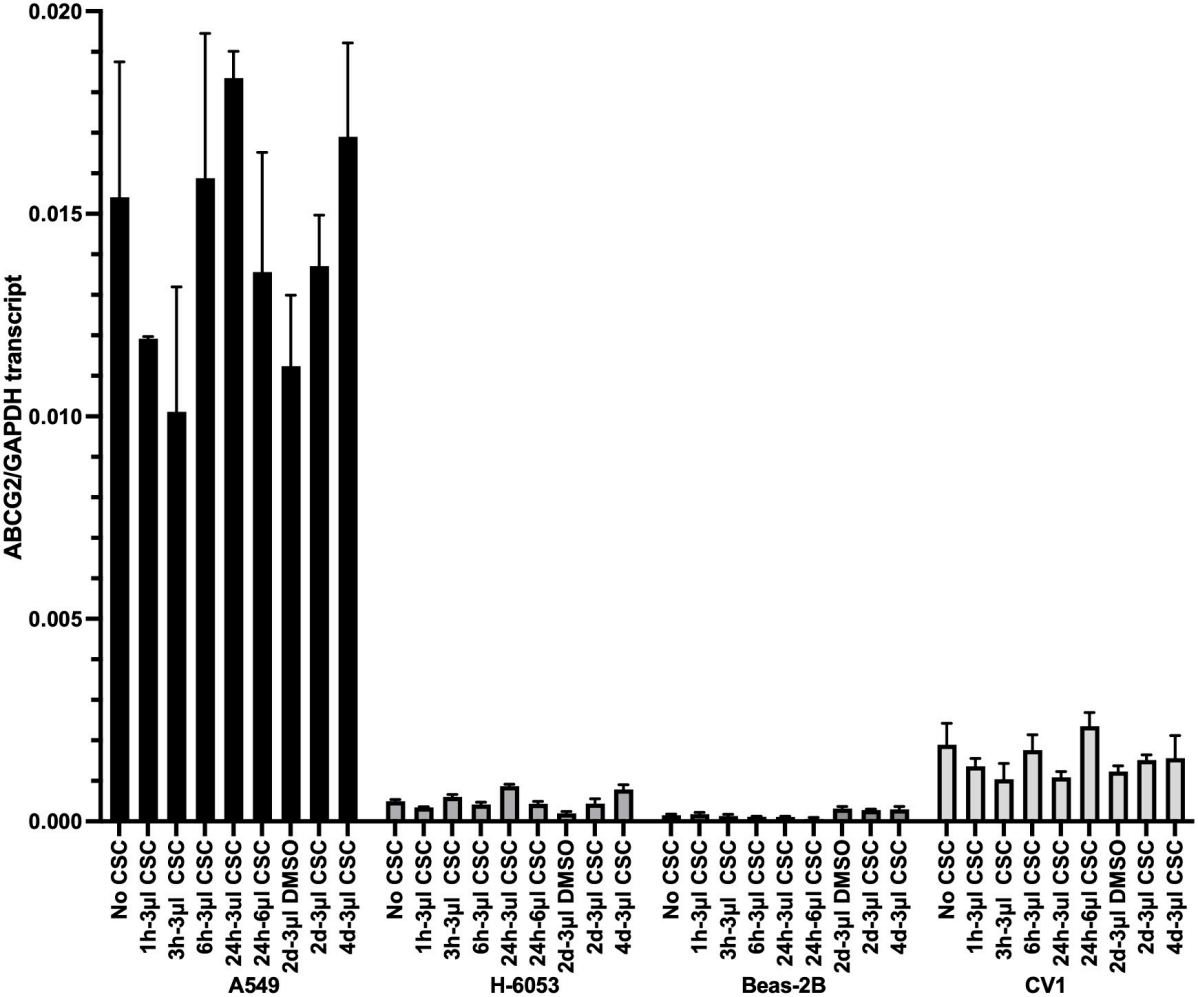
Comparison of the amounts of ABCG2 transcript in different cell lines. The cells were incubated with or without 3 μl CSC per 2 ml of media for 1h, 3h, 6h, and 24 hours, or with 6 μl CSC for 24 hours. Amounts of ABCG2 were analyzed by real time RT-PCR. The amounts of ABCG2 transcript were normalized by the GAPDH transcript levels.

We first decided to confirm that ABCG2 is highly expressed in A549 cells, and also compare its expression to non-cancerous cells, where more protein aggregation with exposure to CSC was observed. To this end, A549, CV1, H-6053, and Beas-2B cells were incubated with or without 3 μl CSC per 2 ml of media for 1, 3, 6, 24 hours, 2 days, or 4 days, or with 6 μl CSC for 24 hours. As controls, we used the cells that were incubated for 2 days with DMSO, and the cells that were untreated (no CSC). Then, the messenger RNA levels of the *ABCG2* transcript were compared using real time RT-PCR, with GAPDH as a normalization control. We detected substantially higher levels of *ABCG2* in A549 than in either of the non-cancerous cell lines, both with and without CSC exposure (Fig 5). The amount of transcript did not change that much during the 4 days observation time. Longer observation was not possible at this CSC concentration since that the normal cell lines deteriorated.

We also analyzed the protein expression of ABCG2 in different cell lines with Western blot (S2 Fig). Since ABCG2 is a transmembrane protein, we had to use a specially formulated buffer (see Material and methods) to prepare protein extracts. The amount of ABCG2 remained roughly the same in A549 cells, incubated with 3 μl of CSC for 1h, 3h, 6h, 24 hours as in the untreated control, which is in accordance to the similar levels of transcript in those conditions. However, we observed some increase of ABCG2 relative to GAPDH upon incubation for 24 hours with 6 μl CSC, even though there was no increase detected in transcript level. This difference can be due to an increase in ABCG2, but it cannot be excluded that it was caused by an increased isolation of ABCG2 from cells that were exposed to higher concentration of CSC, which can potentially affect the integrity of the cellular membrane.

### Inhibition of ABCG2 in A549 cell line with febuxostat resulted in increased protein aggregation

Knowing that ABCG2 is highly expressed, and its substrates represent a wide variety of aromatic molecules, we hypothesized that ABCG2 can participate in reducing the effects of smoke components on aggregation in A549 cells.

**Fig 6:**
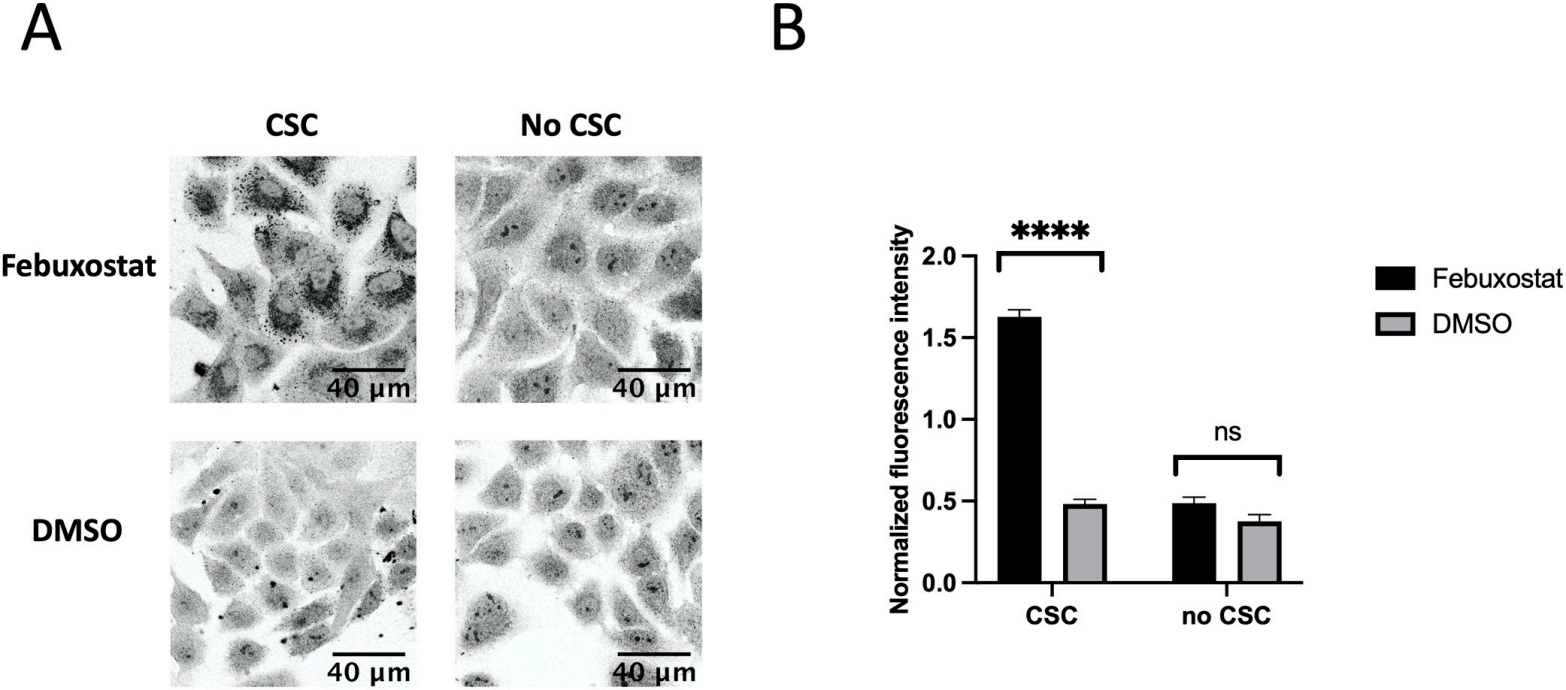
Increase of protein aggregation in A549 cells upon inhibition of ABCG2 with 1 μg/ml febuxostat. A. Cells were treated with 0.3 μl of CSC per 2 ml of media or the same amount of DMSO. B. NFI of the protein aggregates shown in A.

To test the role of ABCG2 in protein aggregation, we used a known inhibitor of ABCG2, febuxostat [35]. A549 cells were incubated with 1 μg/ml febuxostat for 20 hours, followed by the exposure of treated cells to 0.3 μl of CSC. Upon febuxostat treatment, we observed a significant increase in protein aggregation, corresponding to about 3 times increase in NFI (Fig 6A and 6B). Febuxostat treatment in the absence of CSC did not cause significant protein aggregation. This result most likely indicated that inhibition of ABCG2 resulted in an increase of protein aggregation, however, we wanted to confirm this with an independent method.

### Downregulation of expression of ABCG2 with small hairpin RNA resulted in increased protein aggregation in A549

**Fig 7:**
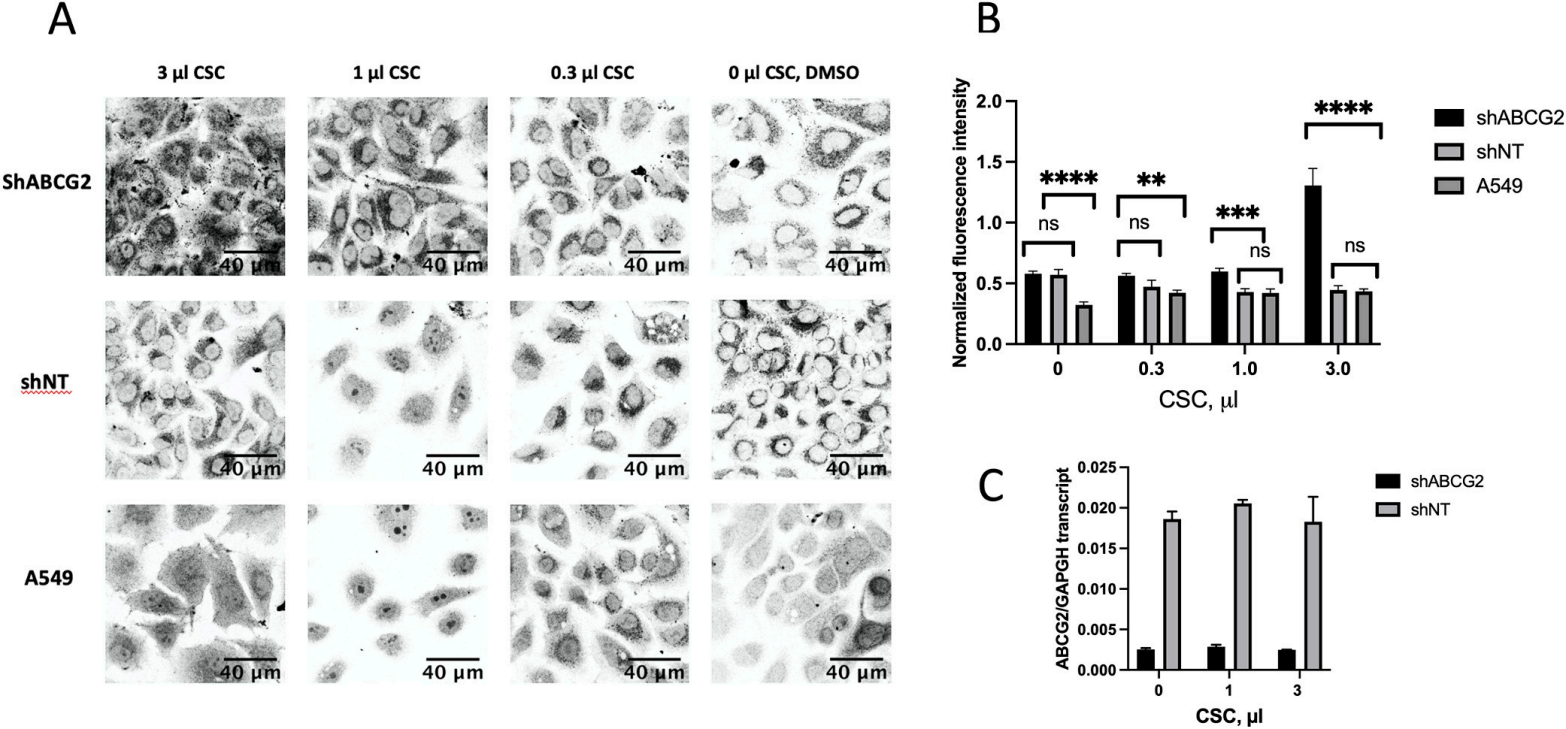
Knockdown of ABCG2 in A549 resulted in increased protein aggregation. A. CSC-induced protein aggregates in knockdown (shABCG2) and control (shNT, and A549) samples, stained by Proteostat Aggresome Detection kit. B. NFI of the protein aggregates shown in A. C. The real time RT-PCR with primers corresponding to ABCG2, showing the efficiency of the knockdown with or without CSC exposure for 24 hours; GAPDH was used for normalization.

To perform a more targeted inhibition of ABCG2, we downregulated it by using a short-hairpin RNA (shRNA) delivered into A549 cells by a lentivirus. To this end, we transduced A549 with lentiviruses expressing shRNAs, complementary to *ABCG2*, or a non-targeting shRNA as a control. Since viral infection itself can induce protein aggregation [36], we incubated our cells 4 days after viral infection before adding CSC, so that the level of aggregation due to the viral exposure subside. Some residual protein aggregation could still be observed in controls that were treated with the virus, but not exposed to CSC (Fig 7A and 7B). However, after CSC exposure, protein aggregation was significantly increased in the cells that expressed shRNA, vastly exceeding the background level, caused by a virus carrying the non-targeting control shRNA. Efficiency of the downregulation was estimated from real time RT-PCR to be (90 ±8) % (Fig 7B), which roughly corresponded to the (90±1) % transduction efficiency observed in A549 (S3 Fig). It was not affected by the presence of CSC in the media at the concentrations that we studied. Upon one day of CSC exposure, the degree of protein aggregation in A549 cells that expressed the shRNA significantly increased, which was reflected in about three-fold increase in the NFI of the cells. This increase in aggregation was similar to what we observed with inhibition of ABCG2 with febuxostat, which implies that ABCG2 was involved in reducing protein aggregation due to CSC.

## Discussion

In this study, we demonstrated a decreased level of protein aggregation and increased survival and growth in the presence of CSC for A549 cancer cells compared to the non-cancerous lung cell lines. We found an experimental support for the hypothesis that ABCG2 plays a role in preventing protein aggregation caused by CSC in adenocarcinoma cell line A549, most likely via efflux of toxic compounds and/or smoke induced radicals. We demonstrated that global protein aggregation was reduced in A549 cells relative to non-tumorigenic Beas-2B and H-6053 lung cells upon CSC exposure, and that the degree of aggregation could be rescued upon perturbing the activity and expression of the ABCG2 transporter protein.

Our first ABCG2 inhibition method utilized febuxostat. Febuxostat is widely used in clinical practice to lower level of uric acid in patients with gout [37]; its clinical effect has been attributed to its ability to interfere with the urate transport function of ABCG2 [35]. The advantage of using febuxostat over other ABCG2 inhibitors was in its low toxicity to humans [37]; in our studies, we have not observed significant levels of protein aggregation associated with febuxostat alone.

Inhibition of ABCG2 with febuxostat resulted in a significant increase in aggregation granules after CSC exposure; the effect can be quantitatively described as about three times increase in the NFI comparing to A549 with active ABCG2. Considering that the aggresomes occupied only a small proportion of the cytoplasm, this moderate increase value was reflecting a substantial increase in aggregation granules.

We validated that ABCG2 was responsible for the reduction of protein aggregation in A549 cells upon CSC exposure by inhibiting its expression using RNA interference. We were able to achieve about 90% downregulation in the expression of ABCG2, and saw an increase in protein aggregate accumulation within the cytoplasm of A549 cells, similar to what was observed with febuxostat. Based on these two experiments, we have concluded the ABCG2 efflux pump plays a role in mitigating the damage caused by cigarette smoke and toxic cellular byproducts. This role is consistent with the previous findings that the membrane localization and expression of ABCG2 was increased in A549 cells upon exposure to CSC [38].

Interestingly, the downregulation of ABCG2 in A549 cells by RNA interference did not affect protein aggregation at low concentrations of CSC. High levels of CSC (> 3 μl) were necessary to observe significant granules in the cytoplasm. This suggested that there could be other protein channels within the cell that were able to manage low amounts of CSC when ABCG2 was inhibited.

In a previous study that analyzed the expression of 48 known ABC transporters in smokers with and without COPD as well as asthma diagnosis, three of them, ABCB6, ABCC1, and ABCC3, were shown to be upregulated by exposure to cigarette smoke [39]. Their list did not include ABCG2, likely because this study did not include cancer patients, however this points to existence of other channels that can additionally assist in the excretion of CS. There are also other mechanisms that can work in conjunction with efflux pumps to rescue cancer cells, such as autophagy or apoptosis impairment [40, 41].

We also assessed whether the non-cancerous cell line can adapt to CSC over time. We observed some moderate adjustment over the week, however, it didn’t reach the low level of aggregation observed in a cancer cell line. This was consistent with previous data that showed that the level of ABCG2 remained low even with smoke exposure in normal lung cells [20].

Other groups also reported that protein aggregate formation is increased upon exposure to cigarette smoke. Exposure to CS in Alzheimer’s transgenic mouse model led to an increase in characteristic abnormalities, such as tau phosphorylation and amyloidogenesis, a type of protein aggregation [42]. It has also been shown that CS exposure led to an increased accumulation of ubiquitinated proteins in the form of insoluble protein aggregates in retinal pigment epithelial cells [43]. Smoke-induced ubiquitinated protein aggregates have also been observed in human COPD-emphysema lung tissues and in Beas-2B cells [12]. Our study proves the involvement of ABCG2 transporter in reducing protein aggregation in A549, which may be a mechanism involved in survival in some of the lung cancers. It is known that continuing smoking during the cancer treatments worsens the prognosis [44–46], possibly due to the fact that resistance to CS involves some of the same mechanisms, as the development of resistance to drugs, one of those being an increase in ABCG2 [38].

## Supporting information

S1 Fig Proteostat aggresome staining of A549 cells treated with proteasome inhibitor or DMSO as a control. (TIFF)

S2 Fig Amount of ABCG2 protein in different cell lines. A. Western blot of ABCG2 and GAPDG isolated from A549, Beas-2B, H-6053, and CV1 cell lines subjected to different smoke concentrations for indicated periods of time. B. Amount of ABCG2 protein in A549 cells, normalized by GAPDH, and then by the value obtained for the No CSC sample.
(TIFF)

S3 Fig The efficiency of transduction, based on GFP expression from the PLL3.7shABCG2 plasmid, is about (90±1) %. GFP fluorescence is shown in the left; the phase contrast image of the same area is shown on the right.
(TIFF)
