# Supplementary figures and images for: ABCG2 transporter reduces protein aggregation in cigarette smoke condensate-exposed A549 lung cancer cells
Source: PLoS One. 2024 Mar 5;19(3):e0297661. doi: 10.1371/journal.pone.0297661 (PMC10914296; doi:10.1371/journal.pone.0297661)

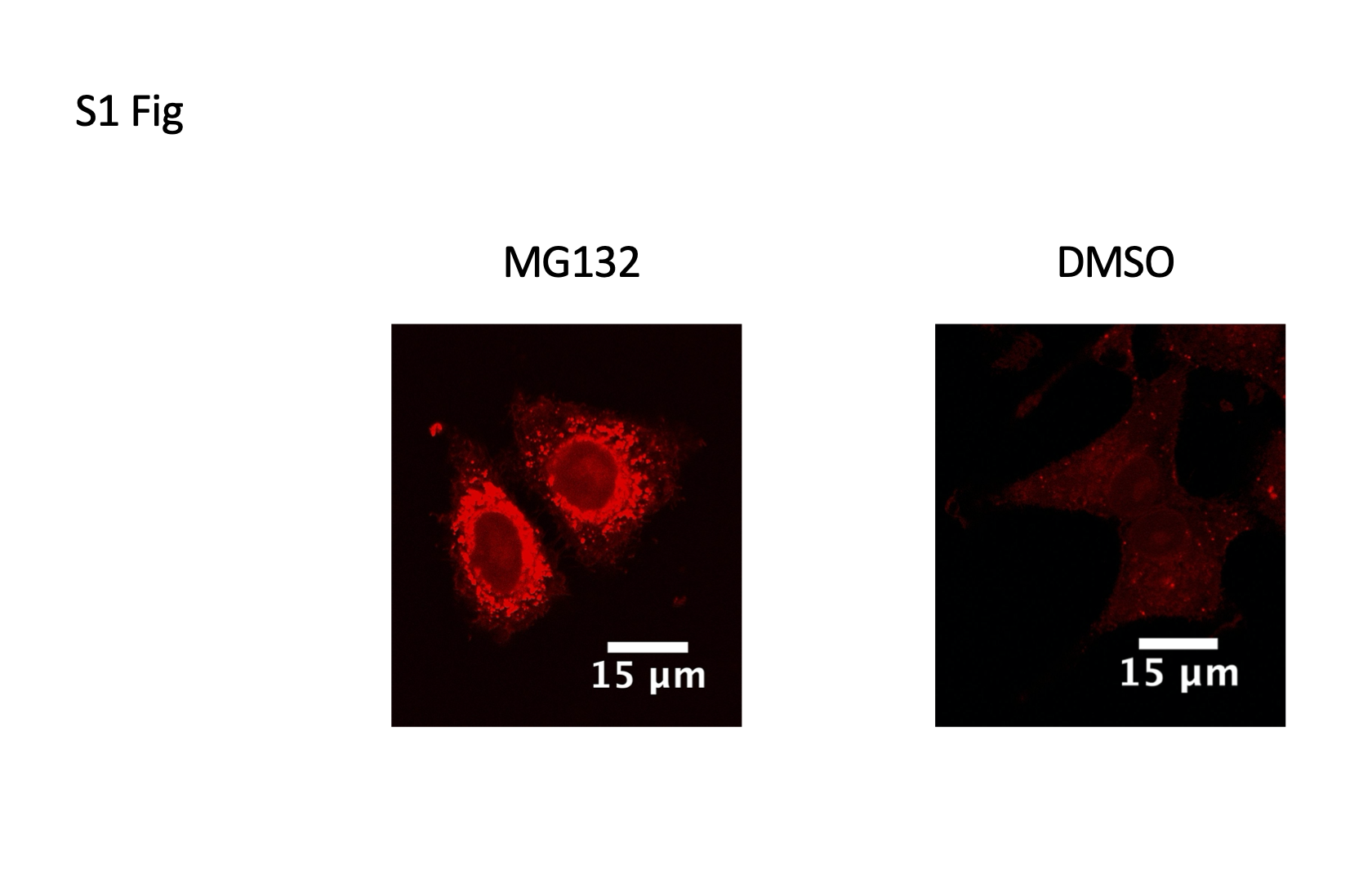

Supplement: S1 Fig — (TIFF) [file pone.0297661.s001.tiff]

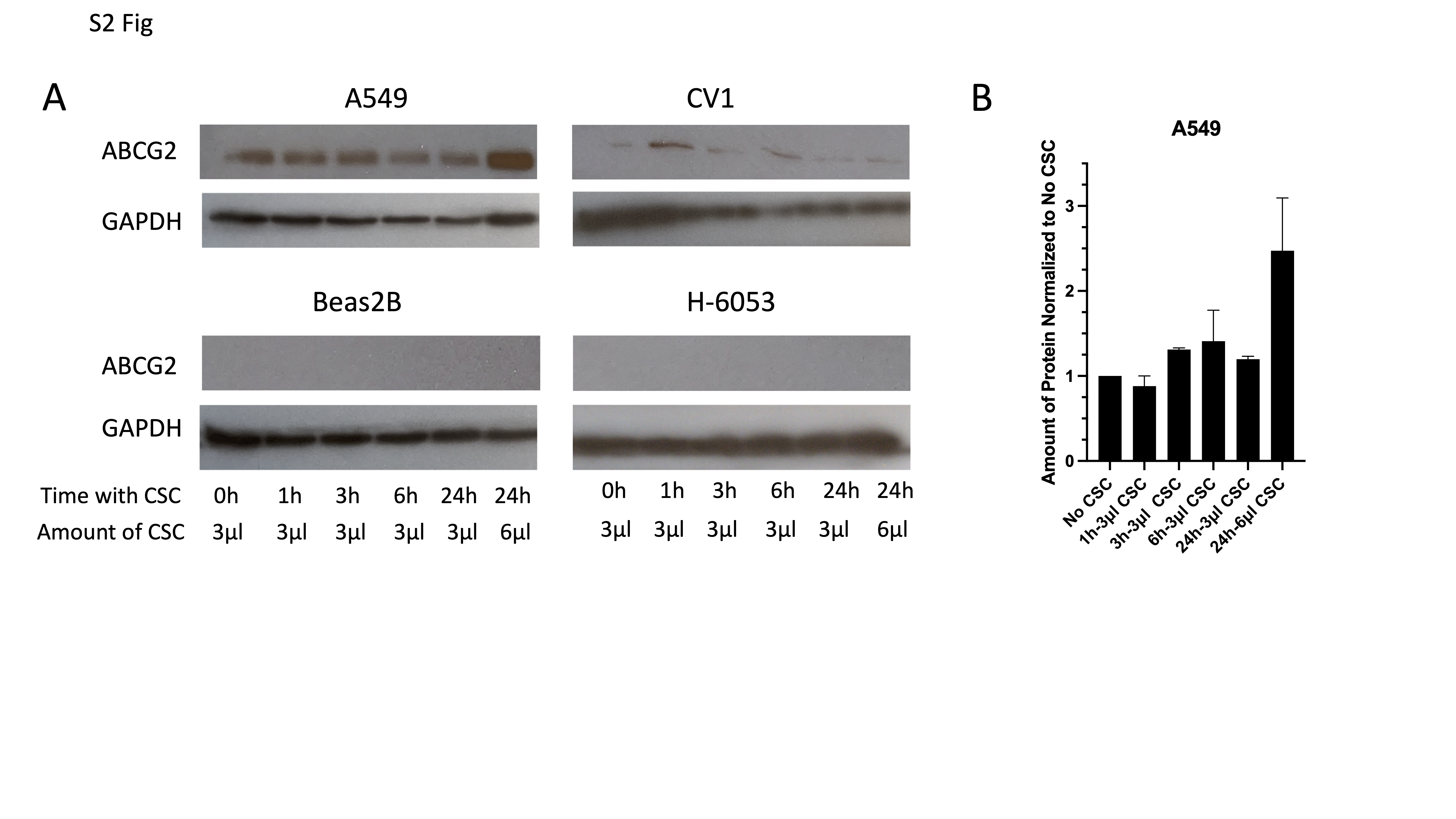

Supplement: S2 Fig — A. Western blot of ABCG2 and GAPDG isolated from A549, Beas-2B, H-6053, and CV1 cell lines subjected to different smoke concentrations for indicated periods of time. B. Amount of ABCG2 protein in A549 cells, normalized by GAPDH, and then by the value obtained for the No CSC sample. (TIFF) [file pone.0297661.s002.tiff]

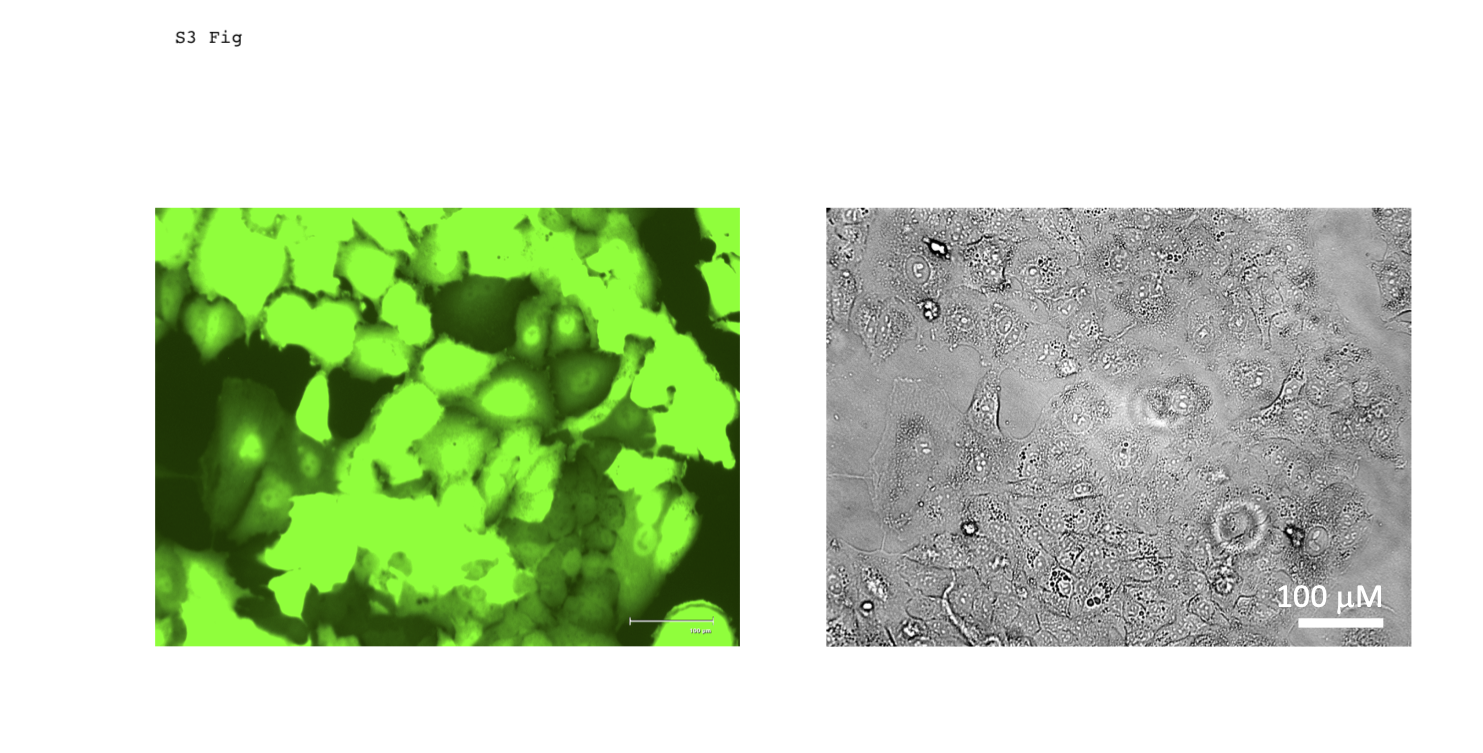

Supplement: S3 Fig — GFP fluorescence is shown in the left; the phase contrast image of the same area is shown on the right. (TIFF) [file pone.0297661.s003.tiff]
